# Impact of a Training Intervention on the Prevention of Aggressions in Nursing Students: A Pre–Post Study

**DOI:** 10.3390/healthcare14121704

**Published:** 2026-06-15

**Authors:** Chaxiraxi Bacallado-Rodríguez, Francisco Javier Castro-Molina, Jesús Manuel García-Acosta, Silvia Elisa Razetto-Ramos, Federico David Bacallado-Rodríguez, José Ángel Rodríguez-Gómez

**Affiliations:** 1 Canary Islands Health Service, 38071 Santa Cruz de Tenerife, Spain; cbacalla@ull.edu.es (C.B.-R.); extjgarciaa@ull.edu.es (J.M.G.-A.); srazetto@ull.edu.es (S.E.R.-R.); 2 Nuestra Señora de Candelaria School of Nursing, University of La Laguna, 38010 Santa Cruz de Tenerife, Spain; 3 Consejería de Educación del Gobierno de Canarias, 38071 Santa Cruz de Tenerife, Spain; fbacrod@gobiernodecanarias.org; 4 Faculty of Nursing, University of La Laguna, 38200 San Cristóbal de La Laguna, Spain; jarogo@ull.edu.es

**Keywords:** workplace violence, aggression, students, education, nursing, nursing education research

## Abstract

**Background:** Violence against healthcare professionals constitutes a global and persistent problem, with significant consequences for professionals’ physical and mental health, organisational climate, and the quality of care provided to patients. **Objective:** To evaluate the impact of a training activity on the prevention and management of aggression in healthcare settings among fourth-year undergraduate nursing students. **Materials and Methods**: The study was conducted at the Nuestra Señora de Candelaria School of Nursing, University of La Laguna. An intervention study with a quasi-experimental design without a control group was conducted through the implementation of a training workshop. A pre- and post-intervention questionnaire was administered to a sample of 59 fourth-year nursing students. In addition, two questionnaires were distributed at the end of the session to assess satisfaction with the training received and students’ self-perceived acquisition of knowledge. This research complied with the TREND statement. **Results:** Descriptive analysis showed higher post-test scores than pre-test scores. The Wilcoxon test indicated a statistically significant difference between the pre-test and post-test scores. Mean knowledge scores increased from 3.16 to 7.58 following the intervention, with a statistically significant difference (*p* < 0.001) and a very large effect size (r = 0.87). **Conclusions:** The training workshop was associated with a significant immediate improvement in knowledge, high levels of satisfaction, and enhanced self-perceived learning.

## 1. Introduction

Violence against healthcare professionals constitutes a global and persistent problem, with significant consequences for professionals’ physical and mental health, organisational climate, and the quality of care provided to patients [1,2,3]. According to the World Health Organization (WHO), more than 50% of healthcare workers experience some form of violence in the workplace at some point during their careers [1]. This phenomenon, which includes physical, verbal, and psychological aggression, has been recognised as an occupational risk requiring comprehensive strategies for prevention, management, and institutional support [1,4,5].

International organisations, including the International Labour Organization (ILO), the International Council of Nurses (ICN), the WHO, and Public Services International (PSI), have for over two decades urged the implementation of ‘zero tolerance’ policies towards violence in the healthcare sector and the establishment of specific training programmes aimed at strengthening staff safety and response capacity [1,3,4]. The ILO Convention No. 190 on Violence and Harassment in the World of Work (2019) reinforces this framework by recognising the right to a violence-free working environment and the need to integrate prevention measures into occupational health systems [3].

In Spain, data from the Ministry of Health show an upward trend in reported incidents of aggression against professionals within the National Health System (NHS), with verbal and psychological aggression predominating, although physical assaults have also increased [6]. The National Institute for Safety and Health at Work (INSST) considers workplace violence to be a psychosocial risk of particular relevance in the healthcare sector and has developed guidelines and Technical Prevention Notes to support its management [7,8]. At the European level, the European Agency for Safety and Health at Work (EU-OSHA) has developed multisectoral guidelines to address third-party violence (users, patients, and companions), highlighting training and internal protocols as key tools [9].

Numerous studies have demonstrated the negative effects of workplace violence on nursing staff, including increased stress, burnout, job dissatisfaction, and intention to leave the profession [10,11]. In response, professional organisations such as the Emergency Nurses Association (ENA) and the European Federation of Nurses Associations (EFN) have issued position statements calling for the inclusion of structured training in aggression prevention and management within professional development and educational programmes [12,13]. Similarly, the WHO and the ILO have emphasised the need to protect workers’ mental health through organisational policies and educational programmes that incorporate the prevention of violence and harassment at work [14,15].

Despite this international consensus, a training gap persists within Spanish higher education. The *Libro Blanco del Título de Grado en Enfermería* [White Paper on the Bachelor’s Degree in Nursing], developed by the National Agency for Quality Assessment and Accreditation (ANECA), does not explicitly include competencies related to the prevention, de-escalation, or management of aggression in clinical settings [16]. Although it addresses aspects related to patient safety, communication, and risk management, it omits the specific skills required to anticipate and respond appropriately to incidents of violence during clinical placements or professional practice. This training gap limits the preparedness of future nurses for situations that are increasingly common in healthcare settings [17,18].

Nursing students, particularly those in their fourth year, represent a particularly vulnerable group, as they are actively involved in real clinical environments throughout the academic year, often without sufficient training to manage aggressive behaviour from patients, relatives, or even colleagues [19]. Several studies indicate that training interventions focused on theoretical knowledge (types of violence, risk factors, legal framework, and protocols), practical skills (therapeutic communication, de-escalation techniques, and self-protection), and attitudes (zero tolerance, incident reporting, and self-care) can enhance perceived competence and coping capacity when facing aggression [20,21,22,23,24]. However, evidence regarding the effectiveness of such interventions in Spanish university contexts remains limited and fragmented.

Within this context, the present study aims to evaluate the impact of a training activity on the prevention and management of aggression in healthcare settings among fourth-year undergraduate nursing students, using a pre-test–post-test intervention design. It is hypothesised that, following the training, participants will demonstrate a significant increase in knowledge, greater perceived preparedness, and strengthened self-reported competencies related to prevention, de-escalation, and communication in situations involving violence.

In this regard, this study seeks to provide evidence supporting the inclusion of these competencies in nursing curricula, in line with the recommendations of the WHO, the ILO, the INSST, and the EU-OSHA, thereby contributing to the creation of safer and healthier care environments for both professionals and patients [1,4,8,9].

## 2. Materials and Methods

### 2.1. Study Design

An intervention study with a quasi-experimental pre-test–post-test design without a control group was conducted through the implementation of a training workshop developed at the Nuestra Señora of Candelaria School of Nursing (EUENSC), affiliated with the University of La Laguna (ULL).

This research adhered to the TREND statement (Transparent Reporting of Evaluations with Non-Randomised Designs) [25], considered one of the most widely recommended guidelines for reporting non-randomised intervention studies, as it provides a checklist to ensure transparency and quality in the reporting of such studies [26].

### 2.2. Population and Sample Size

The study population consisted of fourth-year undergraduate nursing students who were undertaking clinical placements exclusively throughout the academic year. A total of 60 students were voluntarily invited to participate, of whom 59 were ultimately included in the study.

The sample size was estimated for a finite population of 60 fourth-year nursing students. Assuming a 95% confidence level, an expected proportion of 50% (*p* = 0.5), and a maximum acceptable error of approximately 7%, the minimum required sample size was 43 participants. This threshold was established to ensure the representativeness of the target population. Only one individual was excluded from the study due to failure to attend the entire training workshop.

### 2.3. Inclusion and Exclusion Criteria

The inclusion criteria established for the study comprised fourth-year undergraduate nursing students enrolled at the EUENSC during the 2025–2026 academic year who voluntarily agreed to participate in the study. Students with incomplete attendance records or whose questionnaires lacked the necessary coding for analysis during data collection were excluded.

### 2.4. Intervention Development

The workshop was organised into two training blocks. Block 1 consisted of a role-play activity followed by a debriefing session. Block 2 included a lecture on the epidemiology of workplace violence, the legal framework, de-escalation strategies, and reporting procedures. The sessions were delivered by the principal investigator, a nursing faculty member in nursing, and the Healthcare Police Liaison Officer, who contributed expertise in institutional response to aggression.

Prior to the commencement of the training intervention, participants completed the pre-test questionnaire and provided written informed consent.

The theoretical–practical session lasted a total of 5 h and was structured into two training blocks with a 30 min break in between. Initially, the intervention began with a role-playing simulation in which one student assumed the role of a nurse and another that of a patient displaying aggressive and/or threatening behaviour. The remaining students were required to take systematic notes in order to subsequently participate in a debriefing session aimed at fostering critical reflection on the aspects observed during the simulated interaction.

Following this initial activity, a lecture-based session was delivered by the principal investigator (a faculty member of the institution) and the Healthcare Police Liaison Officer from the National Police Force. This session focused on raising awareness and training students in the prevention and management of aggression within healthcare settings.

After the break, the second part of the intervention addressed the magnitude of aggression towards healthcare professionals, presenting up-to-date data on incidence and consequences at the national level. In addition, key theoretical concepts were covered, including different types of violence and conflict de-escalation strategies. Particular emphasis was placed on early detection, underreporting, and the need to systematically record and make visible all incidents of violence.

Subsequently, a guided debate was conducted on individual and collective responsibility in the prevention and reporting of aggression. The role-playing exercise was then repeated, followed by a corresponding debriefing session after the theoretical training had been completed.

Finally, the post-test questionnaire was administered and collected, along with instruments assessing perceived knowledge acquisition and overall satisfaction with the training provided.

### 2.5. Data Collection Instrument

For data collection, two ad hoc questionnaires were developed specifically to achieve the study objectives. The knowledge questionnaire was developed following a comprehensive review of the literature and the identification of the main topics covered in the training workshop. The initial version of the questionnaire was reviewed by a panel of experts comprising professionals with experience in nursing education and academic qualifications at the PhD level, as well as master’s degrees in patient safety and the prevention of workplace violence.

The initial 30-item version of the questionnaire was reviewed by an expert panel in two rounds. The experts assessed each item’s clarity, relevance, and appropriateness using a 4-point Likert scale (1 = poor, 2 = low, 3 = good and 4 = excellent). In the first round, the overall Aiken’s V coefficient was 0.73 and 36.7% of the items reached the predefined validity criterion (V ≥ 0.70). After revising the lower-scoring items, a second round was conducted, and consensus was reached on 22 of the 26 items (85%). The overall Aiken’s V coefficient for the final 26-item version was 0.82, indicating adequate content validity. Four items that did not meet the minimum criterion in either round were removed.

The experts also assessed the relevance, clarity, and appropriateness of the items, and their feedback was used to refine the wording and structure of the questionnaire. A pilot test was conducted to ensure comprehensibility and feasibility prior to final administration. In Table 1, the underlined options correspond to the correct answers.

In addition, a questionnaire assessing satisfaction with the training was adapted from an instrument provided by the Official College of Nursing of Santa Cruz de Tenerife (COE).

The questionnaire designed to measure knowledge acquisition (pre-test–post-test) consisted of 26 closed-ended, single-response polytomous questions (Table 1). The response options were designed to be simple and therefore quick to complete, facilitating participation and increasing the response rate. The questionnaire covered different areas of knowledge, including the regulatory framework of the phenomenon, communication and interpersonal skills, de-escalation strategies and conflict management, institutional resources, cooperation with law enforcement agencies, and reporting channels.

Three additional questions addressing sociodemographic variables were included in the pre-test questionnaire in order to describe the participant profile and to analyse potential associations with the workshop outcomes. The variables included were:

- Age: measured in grouped ranges to avoid the collection of identifiable data and thus ensure participant anonymity (18–23 years, 24–29 years, 30–35 years, and >35 years).
- Gender: categorised as male, female, non-binary, and other.
- Healthcare work experience involving contact with patients, companions, and/or relatives.

These variables allowed the sample to be contextualised and facilitated the identification of potential differences in knowledge acquisition according to student profile.

Participants rotated through several clinical practice units during their fourth year, including emergency care, adult intensive care, neonatology, primary care, and ambulance services.

Self-perceived learning was measured using an ad hoc five-item questionnaire (Table 2), designed to assess the subjective perception of knowledge acquisition following the workshop. Responses were recorded on a six-point Likert-type scale (1 = strongly disagree; 6 = strongly agree), with higher scores indicating a more positive perception of learning.

Training satisfaction (Table 3) was assessed using an adapted questionnaire provided by the COE of Santa Cruz de Tenerife, which evaluates students’ perceived quality of the workshop. The instrument consisted of eleven items distributed across four dimensions:

- Methodology and resources (items 1–2): assesses the adequacy of the materials and the usefulness of the methodology employed.
- Professional team (items 3–5): evaluates clarity of language, ability to resolve questions, and coherence with the planned programme.
- Content (items 6–8): analyses relevance, clarity, and usefulness of the content in relation to aggression against healthcare professionals.
- Overall satisfaction (items 9–11): explores overall perceived usefulness, likelihood of recommendation, and satisfaction with the training received.
- Each item was rated using a five-point Likert-type scale, where 1 = never, 2 = almost never, 3 = sometimes, 4 = almost always, and 5 = always. Higher scores indicated a higher level of satisfaction with the training.
- The questionnaire also included open-ended fields for qualitative observations, allowing participants to provide comments on positive aspects, negative aspects, and areas for improvement of the workshop.

#### Scoring of the Knowledge Questionnaire

The 26-item knowledge questionnaire was scored dichotomously, with correct answers assigned a score of 1 and incorrect answers or ‘I do not know’ responses assigned a score of 0. All items were equally weighted. The total number of correct answers was calculated for each participant and converted to a 0–10 scale for analytical and reporting purposes. The same questionnaire was administered before and after the intervention. Missing responses were treated as item-level missing data and excluded from the analysis of the corresponding item; no participant-level imputation was performed.

### 2.6. Data Collection

The educational intervention, delivered in a workshop format, was conducted in October 2025 during the 2025/26 academic year. Data collection took place at the beginning and at the end of the workshop. Prior to commencement, the principal investigator explained the objectives of the study to all participants, emphasising the voluntary and confidential nature of participation.

Subsequently, informed consent was obtained from those who agreed to participate, which in this case included the entire sample.

In order to preserve anonymity and ensure traceability of the data collected in the pre- and post-intervention assessments, an anonymous numerical coding system was implemented. For this purpose, sixty numerical identifiers were printed on paper and placed in an opaque container. Each participant randomly selected one of these numbers, which they retained throughout the research process and used as their identification code on the various self-administered questionnaires, thereby ensuring confidentiality and enabling correspondence between measurements at both time points.

Immediately after signing the consent form and prior to the start of the training, the pre-test knowledge questionnaire was administered.

Similarly, upon completion of the intervention, participants completed the post-test questionnaire, as well as the training satisfaction questionnaire and the self-perceived knowledge acquisition questionnaire. This procedure enabled the matching of pre- and post-intervention data, along with the additional questionnaires, without compromising participant confidentiality.

Open-ended comments were collected to obtain additional descriptive feedback from participants; however, no formal qualitative analysis was conducted. The responses were reviewed descriptively and summarised narratively.

### 2.7. Data Analysis

Once the study data were obtained, they were exported to an Excel^®^ matrix database, to which access was restricted to the researchers involved in the study. The data were analysed using advanced statistical software, IBM SPSS Statistics for Windows, version 29.0 (IBM Corp., Armonk, NY, USA).

The present study was approved by the Medical Research Ethics Committee (CEIm) in July 2025 (approval code: CHUNSC_2025_64).

## 3. Results

### 3.1. Sample Description

Of the total number of students invited to participate (N = 60), 59 took part in the study (*n* = 59), representing a participation rate of 98.2%.

The majority of participants were women (79.66%; *n* = 47), followed by men (16.95%; *n* = 10); 1.69% (*n* = 1) identified as non-binary and a further 1.69% (*n* = 1) as ‘other’.

Regarding age, more than half of the sample was aged between 18 and 23 years (57.63%; *n* = 34), followed by the 24–29 age group (23.73%; *n* = 14), the 30–35 group (3.39%; *n* = 2), and those aged over 35 years (12.25%; *n* = 9).

In relation to previous professional experience, 79.66% (*n* = 47) reported no prior employment in healthcare settings involving contact with patients, relatives, and/or companions, while 20.34% (*n* = 12) reported previous experience in such contexts.

### 3.2. Assessment of Statistical Assumptions: Evaluation of Normality and Homoscedasticity

The Kolmogorov–Smirnov test was applied to assess the normality of the difference in means between pre-test and post-test scores in the sample (*n* = 59). The test statistic was D = 0.146 with a *p*-value of 0.003, indicating that the data distribution differed significantly from a normal distribution.

In addition, the independence of observations was assessed using the Durbin–Watson test in a regression model with age as a predictor of pre-test scores. The obtained value (DW = 2.314) fell within the acceptable range (1.5–2.5), indicating the absence of significant autocorrelation between observations and confirming the independence of the collected data.

Homoscedasticity was assessed using Levene’s test, confirming equality of variances across the different groups analysed. Among age groups, a Levene’s statistic of F = 0.945 (*p* = 0.425) was obtained, with no significant differences in pre-test scores between the different age groups as assessed by ANOVA (F = 2.659; *p* = 0.057; η^2^ = 0.127). Although a slight tendency towards lower scores was observed in the older age groups, this did not reach statistical significance. Homogeneity of variances by gender was also confirmed (F = 0.002; *p* = 0.961), and ANOVA revealed no statistically significant differences between men and women (F = 0.703; *p* = 0.554; η^2^ = 0.037). The ‘other’ and non-binary categories had small sample sizes; therefore, results for these groups should be interpreted with caution. Similarly, homoscedasticity of pre-test scores according to prior professional experience was verified (F = 0.002; *p* = 0.961). No statistically significant differences were found between participants with and without previous experience (F = 0.705; *p* = 0.483; η^2^ = 0.228), confirming baseline equivalence in initial knowledge.

Overall, the analyses indicated homoscedasticity in all cases and the absence of statistically significant differences in pre-test scores according to age, gender, or work experience.

### 3.3. Psychometric Analysis of the Instruments Used

The reliability of the pre-test–post-test questionnaire was assessed using Cronbach’s alpha coefficient (α = 0.586). Although this value falls within the ‘questionable’ range, evidence indicates that it is acceptable for this exploratory educational intervention study, whose purpose is not individual classification but the detection of group-level change [27,28]. In addition, the heterogeneous nature of the knowledge test, which assesses multiple domains, naturally reduces alpha values while maintaining good item discrimination [29,30]. Furthermore, the obtained value exceeds the standards typically accepted for teacher-developed tests [31] and aligns with recommendations for exploratory research [32].

Accordingly, the instrument was considered appropriate for the objectives of the present study. The self-perceived learning questionnaire demonstrated excellent internal reliability (α = 0.89), and the homogeneity of responses, together with the dispersion statistics obtained, supports the robustness and psychometric quality of the instrument.

Similarly, the internal reliability of the training satisfaction questionnaire was excellent (α = 0.87). High mean scores and low dispersion across all items further reinforce the psychometric robustness of the instrument, supporting its suitability for measuring satisfaction perceptions in the study sample.

### 3.4. Inferential Analysis

**Figure 1:**
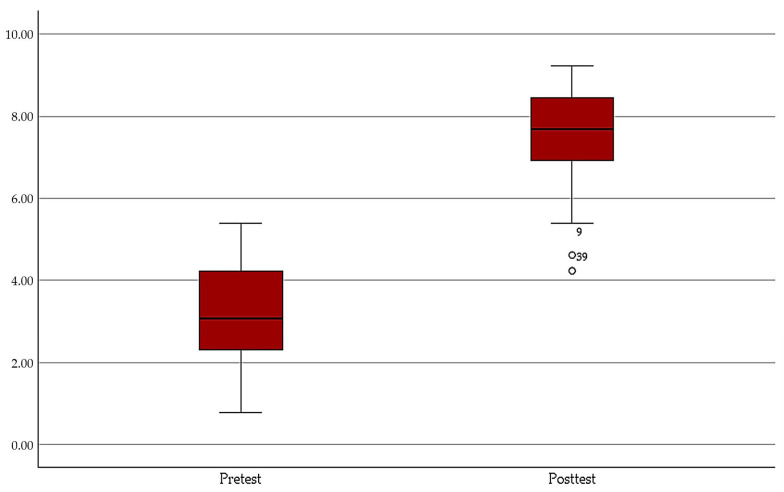
Distribution of knowledge scores (range: 0.0–10) in the pre-test and post-test.

A Wilcoxon signed-rank test was conducted to evaluate significant differences between pre-test and post-test knowledge scores. Descriptive results showed a substantial improvement: the pre-test yielded a mean score of 3.16 (SD = 1.13; range: 0.77–5.38), whereas the post-test achieved a mean score of 7.58 (SD = 1.15; range: 4.23–9.23), representing a mean increase of 4.42 points. The Wilcoxon test indicated a statistically significant difference between the two time points (Z = −6.693; *p* < 0.001). Regarding effect size, a very large effect was observed (r = 0.87), indicating that approximately 76% of the variance in knowledge change (η^2^ = 0.76) was explained by the observed pre–post difference, although causal inference is limited by the study design. Additionally, all participants (100%) showed an increase in their scores, with no decreases or unchanged values recorded (Figure 1).

The percentage of correct responses increased from 31.6% in the pre-test to 75.8% in the post-test, corresponding to an increase in the mean score from 3.16 to 7.58 on a 10-point scale.

**Figure 2:**
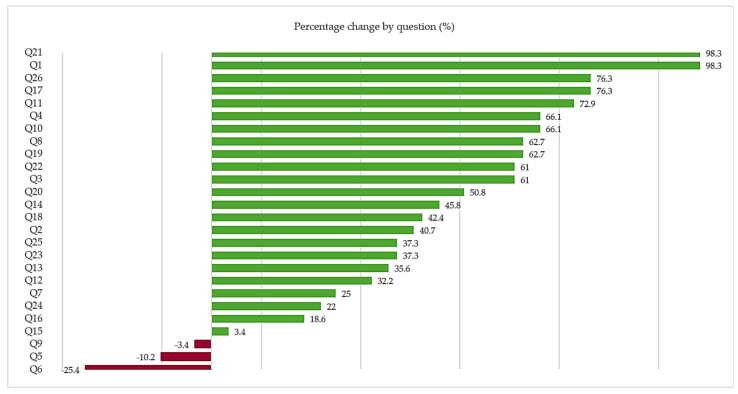
Percentage change per question in correct and incorrect responses on the pre-test and post-test questionnaires (McNemar’s test).

Analysis using McNemar’s test applied to the 26 items of the questionnaire revealed that the educational intervention produced statistically significant changes in 23 out of 26 items (88.5%; *p* < 0.05), with an overwhelming predominance of favourable transitions towards correct responses. Of particular relevance was the identification of eight items (1, 8, 10, 11, 17, 20, 21, 22, and 26) with no instances of regression (b = 0), indicating consolidated learning without erosion of prior knowledge. In addition, a further 13 items showed improvement-to-regression ratios greater than 2:1 (Figure 2).

Items 1 and 21 demonstrated highly consistent improvement, with 58 of the 59 participants showing positive change (χ^2^ → ∞, *p* = 6.94 × 10^−18^), constituting key indicators of fundamental mastery of the competence assessed. In contrast, item 6 exhibited a statistically significant but unfavourable paradoxical pattern (20 regressions vs. 5 improvements; *p* = 0.004), suggesting the need for revision of either its wording or the associated instructional content.

Finally, three items (5, 9, and 15) did not reach statistical significance. This may be attributable to high baseline competence (ceiling effect) or to a lack of differentiated instructional impact. Overall, these findings corroborate the global effectiveness of the intervention at the item level, while simultaneously identifying domains of maximum effectiveness, problematic areas requiring corrective intervention, and opportunities for targeted curricular optimisation.

The decrease observed in Question 6 may be related to the conceptual similarity between response options and the persistence of some residual confusion following the training, despite the overall positive effect of the intervention.

### 3.5. Training Satisfaction

To examine the relationship between overall satisfaction with the training workshop and the variables of age, gender, and prior professional experience, a cross-tabulation analysis and chi-square test were conducted, using item Q3 of the questionnaire, which assesses overall satisfaction, as the reference variable.

With regard to gender, 91.5% of women reported maximum satisfaction, compared with 80% of men, while all non-binary participants and those identifying as other reported maximum satisfaction (several subgroups consisted of a single case). In the analysis by age group, the highest rating predominated across all age ranges, from younger participants (18–23 years: 91.2%) to those aged over 35 years (88.9%), with similar proportions observed in the remaining age groups. Both participants with prior professional experience (91.6%) and those without such experience (89.4%) rated overall satisfaction with the maximum score of 5, showing virtually identical proportions.

Chi-square comparisons revealed no significant relationship between reported overall satisfaction after the workshop and gender (Pearson’s χ^2^ = 0.056; *p* = 0.814), age (Pearson’s χ^2^ = 1.426; *p* = 0.699), or professional experience (Pearson’s χ^2^ = 1.426; *p* = 0.699). The test of independence (Pearson’s χ^2^ = 0.562; *p* = 0.905) also showed no statistically significant association (*p* > 0.05). These findings suggest that the workshop was evaluated positively and homogeneously, with no evidence of structural differences in satisfaction according to the sociodemographic profiles analysed.

### 3.6. Self-Perceived Knowledge Acquisition

In the analysis of self-perceived knowledge acquisition, frequency distributions showed similar patterns across genders, with a predominance of the highest (6) and high (5) ratings, particularly among women (66% rated 6 and 23% rated 5), but also among men and the small groups of other gender identities. The highest rating (6) predominated across all age groups, especially in the main group (18–23 years), where 65% awarded the maximum score. Both participants with prior experience and those without rated the usefulness of the training very highly, with 58% and 41%, respectively, assigning a score of 6.

The chi-square test (Pearson’s χ^2^ = 2.149; df = 3; *p* = 0.542) found no statistically significant association between gender and self-perceived usefulness of the training (*p* > 0.05).

Similarly, no statistically significant association was observed between age and perceived usefulness (Pearson’s χ^2^ = 5.444; df = 9; *p* = 0.794). No significant relationship was found between professional experience and self-perceived usefulness either (Pearson’s χ^2^ = 8.997; df = 9; *p* = 0.438).

In summary, no significant differences were observed in perceived usefulness of the workshop according to gender, age, or professional experience (*p* > 0.05 in all cases). Overall satisfaction was high and homogeneous across all sociodemographic groups. However, these results should be interpreted with caution, as the presence of numerous low expected cell counts may reduce the reliability of the analyses. For future studies, increasing sample sizes within smaller subgroups or using exact tests where appropriate is recommended.

### 3.7. Descriptive Summary of Open-Ended Feedback

Positive and negative aspects were assessed, and areas for improvement identified by the students were explored. Of the 59 students who completed the study, 43 responded to the open-ended questions (72.88%), whereas 16 participants did not provide any comments. Analysis of the open-ended responses regarding overall satisfaction with the workshop revealed a broadly positive evaluation by the participants. Most students highlighted the instructors’ clarity of presentation, the participatory methodology, and the practical relevance of the content, particularly in relation to the prevention and management of aggression in clinical settings.

The workshop dynamics, role-playing activities, and the participation of the invited expert (Healthcare Territorial Police Liaison Officer) were especially well received, as were the approachability of the teaching team and the focus on real-life situations, all of which facilitated understanding and sustained participant interest.

Among the suggested areas for improvement, some students noted the desirability of extending the duration of the session and delving more deeply into practical cases or simulations, with the aim of further strengthening communication and conflict management skills. It was also suggested that additional information on available institutional protocols for dealing with situations of aggression should be included.

With regard to negative aspects, comments were scarce and were mainly related to time constraints or the density of content in certain sections. Overall, the responses reflect a high level of overall satisfaction with the training experience, accompanied by a clear interest in the continuation and expansion of this type of workshop within nursing education.

## 4. Discussion

The results of this study show that nursing students’ knowledge and perceptions regarding aggression in healthcare settings improved significantly following the educational intervention. The observed effect size (r = 0.87) indicates a large pre–post change; however, causal inference is limited by the study design. This finding is consistent with previous evidence suggesting that interactive training programmes can enhance self-confidence and preparedness among healthcare professionals when facing situations of violence [20,21,22,23,24].

The psychometric evaluation of the instruments showed different levels of internal consistency across the questionnaires. The knowledge questionnaire demonstrated moderate internal consistency, which is acceptable for an exploratory educational study aimed at detecting group-level change rather than individual classification. Given the multidimensional nature of the content assessed, this value should be interpreted cautiously and highlights the need for further validation in larger samples. By contrast, the self-perceived learning and training satisfaction questionnaires showed good-to-excellent reliability, supporting their use for descriptive purposes in the present study.

By focusing on preventive training prior to entry into the professional workforce, the present study addresses an emerging need: the early preparation of nursing professionals to cope with workplace violence. The literature highlights that workplace violence in healthcare settings is increasing and has a direct impact on professionals’ physical and psychological health [33]. Accordingly, the results provide evidence in favour of incorporating this type of intervention into undergraduate nursing curricula, equipping students with specific tools to better manage this phenomenon and potentially contributing to a reduction in violent incidents in healthcare environments.

Both overall satisfaction and students’ self-perceived knowledge acquisition following the intervention were high. Participants rated the participatory methodology, relevance of the content, and practical applicability very positively. This aligns with studies indicating that perceived usefulness and contextualisation of training enhance student engagement and facilitate the transfer of learning to clinical practice [26,34]. Suggestions for improvement, such as extending the duration of the sessions or incorporating videos showing real-life cases, provide valuable guidance for optimising future training programmes.

The main limitations of this study include the single-group design, which precludes definitive causal inference; the absence of longitudinal follow-up to assess knowledge retention; and the sample size, which, although sufficient to detect differences in the overall group, may lack adequate power for robust subgroup analyses. In line with this, future research is recommended to adopt controlled designs and to consider the real impact of such interventions on professional behaviour and healthcare safety indicators. With regard to the measurement tools, the robustness of some statistical tests was limited by the presence of a high proportion of cells with fewer than five cases (62.5%), as well as by the fact that 75% of the cells had very low expected counts, which reduces test validity, particularly in small subgroups. Furthermore, the knowledge questionnaire demonstrated moderate internal consistency, which should be interpreted with caution given the heterogeneous and multidimensional nature of the content assessed. The instrument was therefore considered suitable for exploratory educational purposes, although more comprehensive validation using larger samples is recommended. Another limitation is the potential influence of social desirability bias, as the workshop was partly delivered by the principal investigator, which may have influenced participants’ satisfaction ratings and responses regarding perceived learning. Future studies should therefore consider the use of independent evaluators. Finally, as the post-intervention test was conducted immediately after the session, the study assessed only short-term knowledge acquisition and participants’ self-perceived learning. It did not assess long-term retention, the transfer of clinical skills, or behavioural change in real-world practice. The results therefore support the potential educational value of the workshop, but do not demonstrate preparedness to manage real-life situations involving clinical aggression.

From a practical standpoint, the findings suggest that nursing educators should incorporate training interventions addressing aggression into their educational offerings, ideally using active methodologies and simulations to prepare students for real-life scenarios involving violence. Furthermore, healthcare organisations, in collaboration with educational institutions, should integrate prevention and awareness protocols at the student level as part of a coordinated strategy.

In summary, this study provides evidence that an educational intervention specifically designed for nursing students improves their preparedness to manage aggression while also generating high levels of satisfaction. These findings support the need to include early preventive training strategies as an integral component of healthcare education, thereby contributing to the promotion of safer environments for students, professionals, and patients alike.

## 5. Conclusions

The training workshop on aggression prevention for nursing students was associated with a significant immediate improvement in knowledge, increased satisfaction, and enhanced self-perceived learning. The intervention, based on active methodologies and the participation of an Interterritorial Healthcare Police Liaison Officer, demonstrates potential value as an educational tool and should be integrated into undergraduate nursing education. Future research should incorporate a control group and longitudinal follow-up to further strengthen the evidence base.

## Figures and Tables

**Table 1:** Pre-test–post-test knowledge questionnaire.

Questions
**1.** **Which professional group was most affected by aggression in the healthcare sector in Spain in 2024?** a. Physicians b. Nurses c. Administrative staff d. Orderlies e. I do not know
**2.** **What is the most frequent cause of aggression in healthcare settings?** a. Delays in care b. Disagreement with the diagnosis c. User demands d. Lack of respect shown by professionals towards users e. I do not know
**3.** **Which of the following is a non-verbal warning sign of potential aggression?** a. Abruptly and tensely crossing the arms b. Fixed, intimidating stare c. Frowning d. Raising the tone of voice e. I do not know
**4.** **Which law provides prevention and response measures for aggression against healthcare professionals in Spain?** a. General Health Law b. Criminal Code c. Occupational Risk Prevention Law d. Statutory Framework of the National Health System e. I do not know
**5.** **Which is an effective method for managing conflict situations?** a. Avoiding eye contact b. Making rapid and empathetic decisions c. Resolving the conflict collaboratively d. Displaying low authority to reduce tension e. I do not know
**6.** **What primarily characterises assertive communication in a conflict situation?** a. Being direct and respectful b. Being empathetic and manipulative to avoid escalation c. Being authoritarian to avoid showing weakness to the aggressor d. Being passive and maintaining distance for greater safety e. I do not know
**7.** **A basic principle for conflict de-escalation is:** a. Gradually increasing the tone of voice to gain control b. Using open-ended questions c. Using closed-ended responses d. Focusing on one’s own emotions while ignoring the aggressor’s emotions to act safely e. I do not know
**8.** **Which of the following is an effective de-escalation technique?** a. Nodding b. Attempting to increase tension as much as possible in order to then gradually de-escalate it c. Crossing the arms and adopting an alert posture to act quickly d. Sitting silently while the aggressor raises their voice and arms e. I do not know
**9.** **What is the first step in the procedure following an aggression?** a. Reflecting on the incident with colleagues b. Reporting the incident to the service, unit, or primary care team manager c. Calling the police d. Submitting a formal notification using the established form e. I do not know
**10.** **According to the Criminal Code, what type of offence is an aggression against a healthcare professional in the performance of their duties?** a. Minor offence b. Serious offence c. Administrative offence d. Offence of assault against an authority e. I do not know
**11.** **What is the maximum penalty for aggressions causing serious or very serious injuries to a healthcare professional?** a. Prison sentence of 2 to 5 years b. 3 years of imprisonment and community service for 1 year c. There is no maximum penalty established, as it depends on the specific legal context d. There are no aggravating or mitigating circumstances in this type of offence e. I do not know
**12.** **Which information must be mandatorily included in a report of aggression against a healthcare professional according to the Minimum Data Set in Spain?** a. Identity of the aggressor b. Date, location, and type of aggression c. Results of the medical intervention d. Professional registration number of the assaulted professional e. I do not know
**13.** **What is the main purpose of collecting data on aggressions within the system?** a. To penalise the aggressor b. To provide data for future research and safety improvement c. To establish a psychological profile of the aggressor d. To reduce long-term operational costs e. I do not know
**14.** **What is the main objective of the Framework Guidelines for managing aggression against healthcare professionals?** a. To provide free legal advice b. To encourage professionals to resolve aggression individually, quickly, and effectively c. To reduce the costs associated with aggression against healthcare professionals d. To serve as general guidance for addressing workplace violence among healthcare professionals e. I do not know
**15.** **What support measures should be provided to healthcare professionals after an aggression?** a. Financial advice to compensate for loss of income during recovery b. Psychological support and legal advice c. Mandatory transfer to another workplace d. Immediate training in self-defence techniques
**16.** **Which type of aggression is most frequently reported by Spanish healthcare professionals?** a. Physical aggression b. Verbal aggression c. Sexual aggression d. Theft and robbery
**17.** **According to the classification of the California Division of Occupational Safety and Health (Cal/OSHA), which type of violence refers to situations in which there is no prior relationship between the aggressor and the victim and the aggressor may be a recipient of a service provided by the victim?** a. Type I violence b. Violence against healthcare professionals c. Type II violence d. Type III violence e. I do not know
**18.** **What is the main factor that significantly influences healthcare professionals’ decision not to report an aggression?** a. Empathy with the aggressor b. Belief that aggression is inherent to their daily work c. Perceived severity of the aggression d. Gender of the victim e. I do not know
**19.** **Which of the following options corresponds to the pre-alert position?** a. Sitting with hands clasped on the table and both feet flat on the floor b. Standing with the arms raised above waist level, elbows close to the body, and the dominant leg slightly positioned backwards c. Standing with hands visible to the aggressor and the torso slightly leaning forward d. Sitting with arms crossed and feet flat on the floor e. I do not know
**20.** **The reporting rate among healthcare professionals in Spain in 2024 corresponds to:** a. In 2024, 406 reports were filed, of which 106 resulted in arrests b. 4.5% of reported incidents were formally reported, of which 44% were threats and 13.2% were physical injuries c. Approximately 12% of reported aggressions were formally reported, of which 55% were verbal aggressions d. Approximately 15% of reported aggressions were formally reported, of which 25% were physical aggressions e. I do not know
**21.** **The application available in Spain to protect healthcare professionals in contact with law enforcement agencies is called:** a. Segurata Sanitario b. AlertCops c. GCsanit d. There is no application for this purpose in Spain e. I do not know
**22.** **What is the name of the law enforcement role that can act as a mediator during an aggression in a healthcare centre?** a. Healthcare mediator of the Civil Guard b. Healthcare Police Liaison Officer c. Healthcare Police Mediator d. None of the above e. I do not know
**23.** **In which year was the National Observatory on Aggressions created in Spain?** a. 2015 b. 2018 c. 2010 d. None of the above e. I do not know
**24.** **Which of the following strategies is considered effective for the prevention of aggression in a healthcare setting?** a. Implementation of clear and visible security protocols for professionals b. Continuous staff training on conflict management and effective communication with patients c. Use of rapid alert systems to notify facility security when potentially dangerous situations are detected d. All of the above e. I do not know
**25.** **According to conflict de-escalation strategies, which of the following is considered a calculated action?** a. Remaining calm b. Avoiding eye contact c. Placing hands in uniform pockets d. All of the above are incorrect e. I do not know
**26.** **The profile of the victim of aggression in the healthcare sector is:** a. Woman aged 20–30 years b. Woman aged 35–55 years c. Man aged 30–40 years d. None of the above e. I do not know

**Table 2:** Self-perceived learning questionnaire.

Questions
**1. I feel confident that I have acquired new knowledge through this training.** 1 2 3 4 5 6
**2. I consider that the training content was relevant to my professional practice.** 1 2 3 4 5 6
**3. I am confident that I will be able to apply the knowledge acquired during the training to my daily work.** 1 2 3 4 5 6
**4. I would recommend this training to other healthcare professionals.** 1 2 3 4 5 6
**5. I consider that the training was useful in improving my professional competence.** 1 2 3 4 5 6

**Table 3:** Training satisfaction questionnaire.

**Methodology and Resources**
**1. Were the materials and resources used appropriate for the sessions delivered?** 1 2 3 4 5
**2. Did the resources and methodology applied help with understanding the topics covered?** 1 2 3 4 5
**Professional team**
**3. Did the instructors use clear and understandable language?** 1 2 3 4 5
**4. Did they adequately address any questions or doubts?** 1 2 3 4 5
**5. Did they adhere to the programme outlined?** 1 2 3 4 5
**Content**
**6. Was the content aligned with your needs and expectations?** 1 2 3 4 5
**7. Was the content clear and easy to understand?** 1 2 3 4 5
**8. Did the training help you increase your knowledge about aggression against healthcare professionals?** 1 2 3 4 5
**Overall satisfaction**
**9. Did you find this training useful?** 1 2 3 4 5
**10. Would you recommend this training to your colleagues?** 1 2 3 4 5
**11. Overall, are you satisfied with the training received?** 1 2 3 4 5
**Overall, and in relation to the training received, please indicate positive aspects, negative aspects, and areas for improvement.**

## Data Availability

The data presented in this study, as well as the pre-registration details, are available from the corresponding author upon reasonable request.

## Abbreviations

The following abbreviations are used in this manuscript:

WHO	World Health Organization
ICD	International Classification of Diseases
PSI	Public Services International Federation
NHS	National Health System
INSST	National Institute for Safety and Health at Work
EU-OSHA	European Agency for Safety and Health at Work
ENA	Emergency Nurses Association
EFN	European Federation of Nurses Associations
ANECA	National Agency for Quality Assessment and Accreditation
EUENSC	Nuestra Señora of Candelaria School of Nursing
ULL	University of La Laguna
COE	Official College of Nursing
CEIm	Medical Research Ethics Committee
